# Endogenous* Candida* Endophthalmitis as a Rare Complication of Trans-Urethral Lithotripsy in a Healthy Woman: A Case Report

**DOI:** 10.4274/tjo.galenos.2019.02328

**Published:** 2019-10-24

**Authors:** Mohammad Shirvani, Shahla Hosseini, Mehrnoosh Maalhagh, Sahar Mohaghegh

**Affiliations:** 1 Shiraz University of Medical Sciences, Department of Ophthalmology, Shiraz, Iran

**Keywords:** Endogenous endophthalmitis, Candida endophthalmitis, lithotripsy, ureteral stone

## Abstract

Endogenous endophthalmitis is a serious sight-threatening ocular emergency that usually occurs in patients with serious underlying risk factors. In this report, we describe a case of endogenous *Candida* endophthalmitis following trans-urethral lithotripsy in an immunocompetent woman. In our case, the retinal lesion regressed completely and vision was restored. We discuss diagnostic procedures and management strategies in this article.

## Introduction

Endogenous endophthalmitis is an ocular emergency that can lead to catastrophic ophthalmic complications. Endogenous fungal endophthalmitis (EFE) results from dissemination of fungal organisms from infected organs to the ocular vascular network following fungus seeding in the choroid and retina.^1,2,3^ The organisms responsible for EFE are *Candida, Aspergillus*, and Coccidious.^2,3^ Trans-urethral lithotripsy (TUL) is a minimally invasive endoscopic procedure performed using a rigid or flexible uretroscope.^4^ Here, we report a rare case of endogenous *Candida* endophthalmitis (ECE) after TUL in a healthy woman.

## Case Report

**Figure 1:**
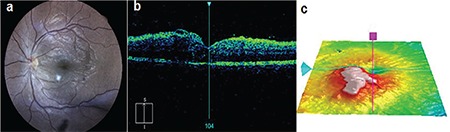
Initial appearance at time of presentation. (a) Color fundus photo showed a creamy lesion in the parafoveal area; (b) Spectral-domain optical coherence tomography revealed macular edema and micro-abscess formation in the sensory retina; (c) Topographic macular map displayed an elevated lesion on the macula

A 31-year-old woman presented to the ophthalmology emergency room complaining of painless, gradual reduction in visual acuity in her left eye starting 1 week earlier. The patient had undergone TUL with double-J stent placement for a 19-mm proximal left ureteral stone 2 weeks before presentation to the ophthalmology clinic. Past medical and drug history was negative. Pre- and postoperative urine and blood cultures were negative and urine analysis was unremarkable. Upon examination, her best-corrected visual acuity (BCVA) in the left eye was 1/10. Intraocular pressure was 11 mmHg. Slit-lamp examination revealed +1 ciliary injection with no signs of keratic precipitate (KP), and hypopyon and +1 cells in the anterior chamber. The iris and lens were normal. Mild vitritis was seen in the vitreous cavity. On fundus examination, media was clear and a creamy, mildly elevated lesion 1/4 disc diameter in size with indistinct borders was observed in the inferior parafoveal region (Figure 1a). Spectral-domain optical coherence tomography showed subretinal fluid aggregation and macular edema (Figure 1b, c). Examination of the right eye was unremarkable.

Following hospital admission, a diagnostic vitreous tap was performed and a sample was sent for smear, culture, and real-time polymerase chains reaction (RT-PCR) analysis. The smear was unremarkable, but RT-PCR was positive for *Candida albicans*. Therefore, intravitreal injection of amphotericin-B (0.5 µg/0.1 mL) was performed. Treatment with topical levofloxacin, hematropin, and prednisone acetate 1% every 6 hours and oral fluconazole 200 mg every 12 hours was initiated. Blood and urine culture at the time of presentation were negative and urine analyses were unremarkable. Viral markers including hepatitis B virus surface antigen and core antibody, hepatitis C virus antibody, and human immunodeficiency virus antibody (HIV Ab) were negative. Serology was negative for *Toxoplasma* (IgM and IgG), *Borrelia*, and *Bartonella*. In systemic workup, antinuclear antibody, antineutrophil cytoplasmic antibody, antimitochondrial antibody, venereal disease research laboratory, fluorescent treponemal antibody absorption, Mantoux, and interferon-γ tests were all negative. Erythrocyte sedimentation rate, C-reactive protein, complete blood count, platelet count, fasting blood glucose, aspartate aminotransferase, alanine aminotransferase, blood urea nitrogen, creatinine, angiotensin converting enzyme, and immunoglobulin G, M, and A levels were within normal limits. Peripheral blood smear and paranasal sinus and chest x-rays were normal.

Forty-eight hours after initiating treatment, the patient’s BCVA increased to 3/10. Conjunctival injection and vitritis disappeared, and the borders of the infiltrative lesion became sharp. She was discharged with oral fluconazole 200 mg every 12 hours for 6 weeks. Vitreous tap culture was negative after 72 hours.

**Figure 2:**
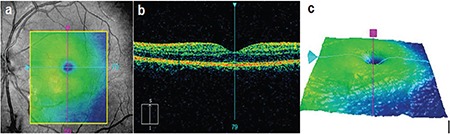
Regression of the fungal lesion 6 weeks after antifungal treatment. (a) Infrared fundus image showed complete disappearance of the fungal lesion; spectral-domain optical coherence tomography (b) and topographic macular map (b) revealed resolution of the macular edema without scarring or traction formation

After 6 weeks, her BCVA was 9/10 and the fungal infiltrative lesion had completely disappeared. Macular edema was resolved with no scarring or epiretinal membrane formation (Figure 2a, b, c). The final BCVA outcome was 10/10 and there was no recurrence in 3-year follow-up.

## Discussion

ECE is a devastating ocular infection. Predisposing conditions include long-term systemic antibiotic usage, hospitalization, indwelling catheters, candiduria, major gastrointestinal intervention, prolonged intravenous line, hemodialysis, liver cirrhosis, intravenous drug abuser, immunomodulatory therapy, chemotherapy, diabetes mellitus, hematopoietic, organ transplantation, abortion, and HIV.^1,2,3,5^

Fungi may enter bloodstream during urinary tract interventions due to mechanical abrasion and epithelial trauma, leading to candidemia and intraocular candidiasis. Some reported infectious complications after urinary tract procedures include urinary tract infection, urosepsis and candidemia, perinephric and renal abscesses, urinoma, *Klebsiella* endophthalmitis, and retroperitoneal abscess.^6^ We found 5 case reports of ECE following urinary tract lithotripsy in our literature review.^7,8,9,10,11^ In 3 cases, ECE occurred after ESWL and uretroscopy for double-J stent placement.^7,8,9^ In one case, ECE occurred following TUL and ureteral stent placement^10^ and in the last case report it occurred after decompressive nephrostomy.^11^ In 4 cases, preoperative urine culture was positive for *C. albicans* and the patients suffered from debilitating diseases (liver cirrhosis, rheumatic arthritis, alcoholic liver disease, or diabetes mellitus).^8,9,10,11^ In our case, ECE occurred in an immunocompetent woman after TUL double-J stent placement while pre- and postoperative urine and blood cultures were negative and there were no underlying risk factors.

The diagnosis of ECE is difficult due to its various ocular manifestations and low positive culture rate, especially in cases with minimal vitreous involvement. The condition does not only occur in patients with underlying risk factors, but also in healthy individuals. Thus, there is the risk of misdiagnosis, leading to delay in initiating appropriate treatment. For more accurate diagnosis, vitreous tap sampling or diagnostic vitrectomy is recommended in suspicious cases, since diagnostic vitrectomy shows a higher positive culture rate and intravitreal injection can be performed simultaneously.^1,2,3,5,8^ Moreover, RT-PCR is more sensitive than culture, but more expensive and might be unavailable.^1,2,3^ In this case report, RT-PCR analysis of the vitreous sample was positive for *C. albicans*, but vitreous smear and culture were negative.

Timely diagnosis and rapid antifungal therapy are associated with better visual outcomes.^2,3^ ECE treatment depends on the severity of inflammation and the patient’s visual acuity. Appropriate treatment in patients with isolated choroidoretinitis is systemic medication with good intravitreal penetration, such as voriconazole and fluconazole. When a patient presents with choroidoretinitis and mild to moderate vitritis, systemic therapy accompanied by intravitreal injection of amphotericin-B or voriconazole is appropriate. In sight-threatening conditions and severe vitritis, pars plana vitrectomy with intravitreal medication during vitrectomy and systemic medication are recommended.^1,2,3^ Although intravitreal injection of amphotericin-B is very effective, intravenous injection of amphotericin-B is not recommended due to poor intravitreal penetration and systemic complications such as nephrotoxicity.^1^ In our case, swift diagnosis and appropriate antifungal treatment (systemic fluconazole + intravitreal amphotericin-B) led to good visual outcome.

ECE after urinary tract interventions is a rare but vision-threatening infection that may occur in immunocompetent individuals. Early detection and timely treatment can lead to better visual prognosis.
